# miR-155-5p inhibition promotes the transition of bone marrow mesenchymal stem cells to gastric cancer tissue derived MSC-like cells via NF-κB p65 activation

**DOI:** 10.18632/oncotarget.7767

**Published:** 2016-02-26

**Authors:** Mengchu Zhu, Mei Wang, Fang Yang, Yiqing Tian, Jie Cai, Huan Yang, Hailong Fu, Fei Mao, Wei Zhu, Hui Qian, Wenrong Xu

**Affiliations:** ^1^ Key Laboratory of Medical Science and Laboratory Medicine of Jiangsu Province, School of Medicine, Jiangsu University, Zhenjiang, Jiangsu, China

**Keywords:** mesenchymal stem cells, gastric cancer, microRNA, tumor microenvironment

## Abstract

Gastric cancer tissue-derived MSC-like cells (GC-MSC) share similar characteristics to bone marrow MSC (BM-MSC); however, the phenotypical and functional differences and the molecular mechanism of transition between the two cell types remain unclear. Compared to BM-MSC, GC-MSC exhibited the classic phenotype of reactive stroma cells, a stronger gastric cancer promoting capacity and lower expression of miR-155-5p. Inhibition of miR-155-5p by transfecting miRNA inhibitor induced a phenotypical and functional transition of BM-MSC into GC-MSC-like cells, and the reverse experiment deprived GC-MSC of tumor-promoting phenotype and function. NF-kappa B p65 (NF-κB p65) and inhibitor of NF-kappa B kinase subunit epsilon (IKBKE/IKKε) were identified as targets of miR-155-5p and important for miRNA inhibitor activating NF-κB p65 in the transition. Inactivation of NF-κB by pyrrolidine dithiocarbamic acid (PDTC) significantly blocked the effect of miR-155-5p inhibitor on BM-MSC. IKBKE, NF-κB p65 and phospho-NF-κB p65 proteins were highly enriched in MSC-like cells of gastric cancer tissues, and the latter two were correlated with the pathological progression of gastric cancer. In GC-MSC, the expression of miR-155-5p was downregulated and NF-κB p65 protein was increased and activated. NF-κB inactivation by PDTC or knockdown of its downstream cytokines reversed the phenotype and function of GC-MSC. Taken together, our findings revealed that miR-155-5p downregulation induces BM-MSC to acquire a GC-MSC-like phenotype and function depending on NF-κB p65 activation, which suggests a novel mechanism underlying the cancer associated MSC remodeling in the tumor microenvironment and offers an effective target and approach for gastric cancer therapy.

## INTRODUCTION

Tumor associated stroma cells are important components of the tumor microenvironment and have been found to play a role in cancer progression [1, 2]. Stroma cells include fibroblasts, endothelial cells, mesenchymal stem cells (MSCs), pericytes and infiltrating immune cells. Accumulating evidence indicates that these cells originate from normal cells but become altered during tumor development [2]. During cancer invasion and metastasis, quiescent resident fibroblasts in the normal stroma can be transformed into cancer-associated fibroblasts (CAFs) and become the most prominent cell type within the tumor stroma of many cancers [2, 3]. In contrast to normal fibroblasts, CAFs have phenotypical and functional abnormalities. CAFs express myofibroblast markers, secrete distinctive cytokines and extracellular matrix, and have the capacity to facilitate tumor initiation, growth and progression [4, 5, 6].

Our research group was the first to successfully isolate MSC-like cells from gastric cancer tissues and adjacent non-cancerous gastric tissues, which were designated as gastric cancer tissue-derived MSC-like cells (GC-MSC) and adjacent non-cancerous gastric tissues-derived MSC-like cells (GCN-MSC), respectively [7, 8]. GC-MSC share similar surface markers and the multi-differentiation potential with GCN-MSC, but secrete higher levels of several inflammatory cytokines, including IL-6, MCP-1, and VEGF, and have a stronger tumor-promoting ability [8, 9, 10]. Bone marrow derived stem cells (BM-MSC) exhibit marked tropism for tumor sites and have the ability of transition into cancer associated stroma cells [11, 12]. However, the phenotypical and functional differences and the molecular mechanism of transition between BM-MSC and GC-MSC have not been systematically studied.

MicroRNAs (miRNAs) are small non-coding RNAs that play an important role in diverse biological processes. They act as oncogenes and tumor suppressors and are involved in many aspects of the behavior of cancer cells [13, 14]. The aberrant expression and regulatory roles of miRNAs are being expanded to the tumor microenvironment [15, 16, 17]. Recent advances have highlighted the important role of miRNAs in microenvironment transformation [18]. Mitra *et al.* showed that miR-241, miR-31, and miR-155-5p directly reprogram normal fibroblasts into CAFs in ovarian cancer [19]. Pang *et al.* found that pancreatic cancer secreted microvesicles reprogrammed normal adjacent fibroblasts into CAF by miR-155-5p [20]. Their studies suggest that miR-155-5p plays an important role in the conversion of normal fibroblasts into CAFs. Whether miR-155-5p is aberrantly expressed in GC-MSC and directly regulates the transition of BM-MSC into GC-MSC remains unclear. Here, we analyzed the phenotypical and functional differences between BM-MSC and GC-MSC, determined miR-155-5p expression levels in GC-MSC versus BM-MSC, and focused on the regulatory role and mechanism of miR-155-5p in the transition of BM-MSC into GC-MSC.

## RESULTS

### Phenotypical and functional differences between BM-MSC and GC-MSC

**Figure 1:**
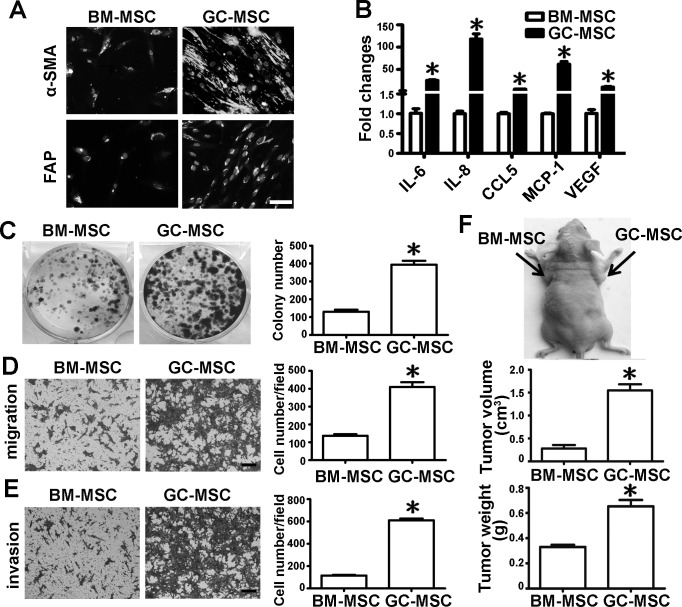
Comparison of cell phenotype and function between BM-MSC and GC-MSC Phenotypical analysis: **A.** α-SMA and FAP protein levels detected by immunofluorescent staining, Magnification: 200×, *Scale bar* = 50μm. **B.** *q*RT-PCR of cytokines mRNA expression; Functional analysis: Conditioned medium were collected from MSCs and used to treat gastric cancer cell line HGC-27. **C.** Colony formation assay. The number of colony was counted and presented as columns. **D.** Migration analysis. **E.** Invasion assay. Magnification: 100×, *Scale bar* =50μm. Representative graphs were shown. Cell number of each field were counted and presented as columns. **F.** *In vivo*, HGC-27 suspended in MSC conditioned medium was subcutaneously injected into the flank of BALB/c nude mice (n = 6 for each group). Tumors were surgically removed 20 days after injection. Representative graph of nude mice bearing tumor, tumor volume and tumor weight are shown. Data were presented as Means ±SD.*, *P* < 0.05.

We successfully isolated MSCs from the bone marrow (BM-MSC) and gastric cancer tissues (GC-MSC). The morphology, cell-surface markers and differentiation potential were the same between BM-MSC and GC-MSC (Supplementary Figure 1). However, their phenotype and function in gastric cancer were significantly different (Figure 1). The immunofluorescent intensity of alpha-smooth muscle actin (α-SMA) and fibroblast activation protein (FAP) as markers for reactive stroma cells were stronger in GC-MSC than in BM-MSC (Figure 1A). Several inflammation-related cytokines including IL-6, IL-8, CCL-5, MCP-1 and VEGF measured by quantitative real-time polymerase chain reaction (*q*RT-PCR) were highly expressed in GC-MSC (Figure 1B). To assess the functional differences between the two types of MSCs in gastric cancer, their cell culture medium were collected to treat gastric cancer cell line HGC-27. Colony formation assays revealed that the number of cell colonies in the GC-MSC group was higher than that in the BM-MSC group (Figure 1C). Transwell migration and invasion analysis showed that the number of migrating and invading gastric cancer cells in the GC-MSC group were more than those in the BM-MSC group (Figure 1D and 1E). *In vivo*, we subcutaneously injected HGC-27 cells mixed with the conditioned medium from MSCs into nude mice to establish a xenograft tumor model. The tumor volume and weight were larger and heavier in the GC-MSC group than in the BM-MSC group (Figure 1F). These data indicate that BM-MSC and GC-MSC share the common characteristics of MSCs, but they show phenotypical and functional differences.

### The role of miR-155-5p in the phenotype of MSC

**Figure 2:**
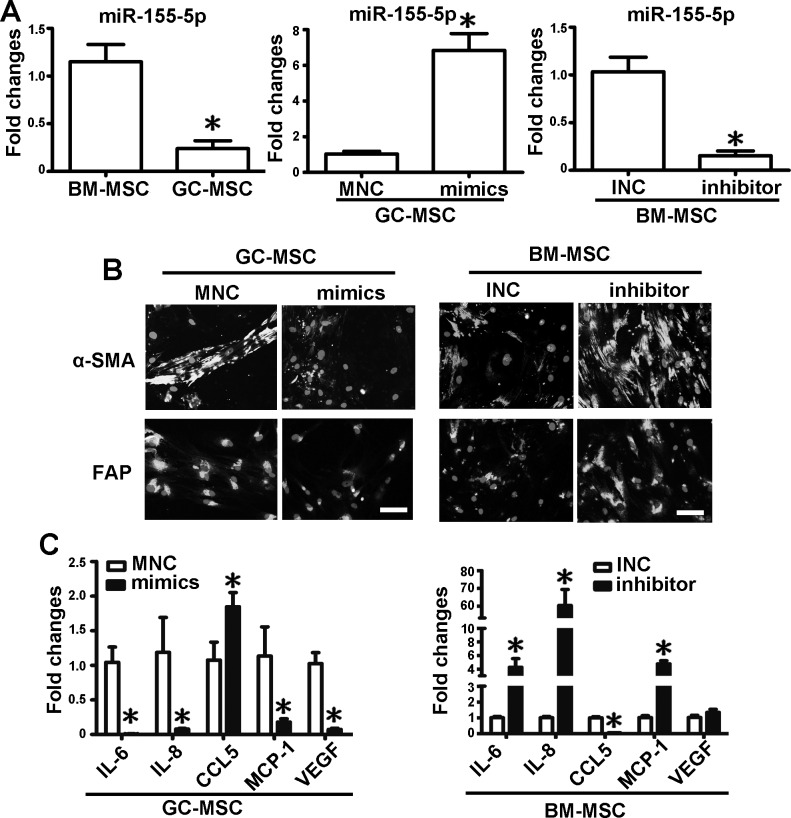
miR-155-5p underexpression confers BM-MSC with GC-MSC-like phenotype **A.** *q*RT-PCR of miR-155-5p expression levels in MSCs. Overexpression of miR-155-5p in GC-MSC by miR-155-5p mimics (mimics). Knock down of miR-155-5p level in BM-MSC by miR-155-5p inhibitor (inhibitor). Mimics negative control (MNC) and inhibitor negative control (INC) were set as corresponding control. **B.** α-SMA and FAP protein levels tested by immunofluorescence. **C.** *q*RT-PCR of cytokines mRNA expression. Data were presented as Means ±SD.*, *P* < 0.05.

*q*RT-PCR analysis showed that miR-155-5p expression level was significantly lower in GC-MSC than in BM-MSC (Figure 2A). To evaluate the role of miR-155-5p in the phenotype of MSC, miR-155-5p was overexpressed by transfection of GC-MSC with miRNA mimics. Mimics negative control (MNC) was set as the control (Figure 2A). A miRNA inhibitor was used to suppress miR-155-5p expression in BM-MSC and inhibitor negative control (INC) was used as the control (Figure 2A). miR-155-5p overexpression reduced the immunofluorescence intensity of α-SMA and FAP in GC-MSC, whereas the immunofluorescence intensity of the two markers in BM-MSC was enhanced by the miR-155-5p inhibitor and was similar to the intensity detected in GC-MSC (Figure 2B). miRNA mimics significantly reduced the expression levels of most of the cytokines, and only increased the level of CCL5 in GC-MSC. By contrast, IL-6, IL-8 and MCP-1 were upregulated and CCL5 was downregulated by the inhibitor in BM-MSC. VEGF expression levels were not changed (Figure 2C). These data suggest that miR-155-5p is downregulated in GC-MSC and is important for sustaining their phenotype. miR-155-5p inhibition causes BM-MSC a phenotype similar to that of GC-MSC.

### Effect of miR-155-5p on the function of MSC in gastric cancer

**Figure 3:**
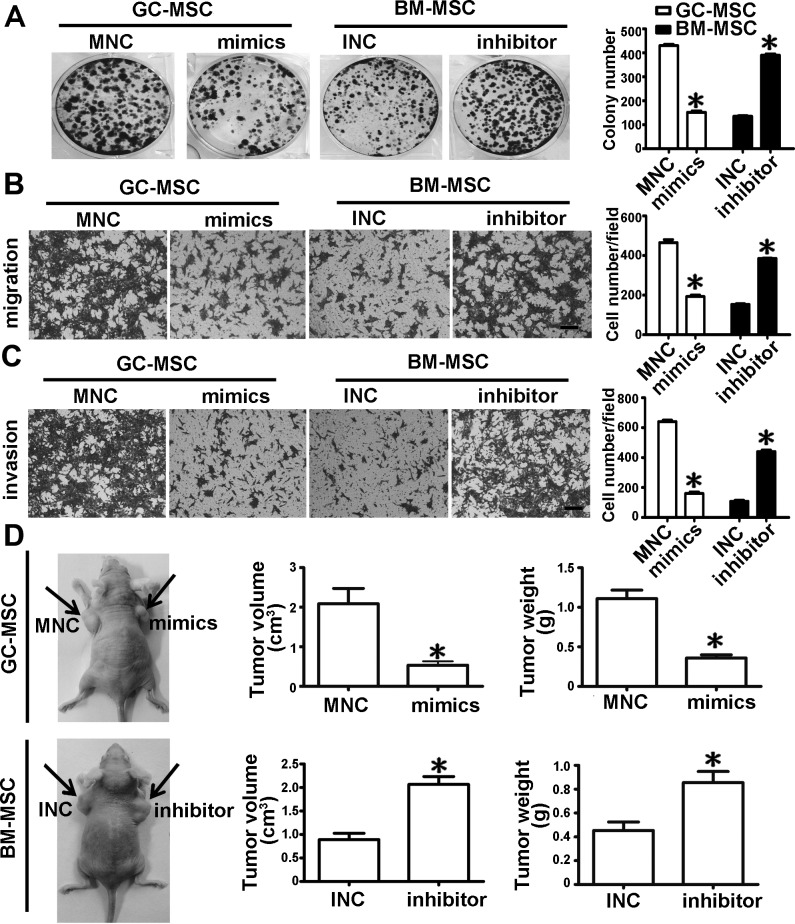
miR-155-5p downregulation promotes BM-MSC to acquire GC-MSC-like function in gastric cancer **A.** Colony formation assay. **B.** Migration analysis. **C.** Invasion assay. **D.** *In vivo*, HGC-27 mixed with MSC conditioned medium was subcutaneously injected into the nude mice (*n* = 6 for each group). Representative graph of nude mice bearing tumor, tumor volume and tumor weight are presented. Data were presented as Means ±SD.*, *P* < 0.05.

We collected the cell culture medium from the above transfected MSCs and treated HGC-27 cells. Colony formation assays showed that the number of cell colonies in the miRNA mimics-transfected GC-MSC group were less than those in the MNC transfected GC-MSC group. Compared to the INC-transfected BM-MSC group, the number of cell colonies was more in the miRNA inhibitor-transfected BM-MSC group, which resembled the MNC-transfected GC-MSC group (Figure 3A). Compared to the corresponding control groups, the migrating and invasive capacities of HGC-27 cells were significantly attenuated in the miRNA mimics-transfected GC-MSC group, but were enhanced in the miRNA inhibitor-transfected BM-MSC group and similar to that in the MNC-transfected GC-MSC group (Figure 3B and 3C). Consistent with the results *in vitro*, the tumor volume and weight were decreased in the miRNA mimics-transfected GC-MSC group (Figure 3D), whereas the tumor volume and weight were increased in the miRNA inhibitor-transfected BM-MSC group *in vivo* (Figure 3D). These data indicate that ectopic expression of miR-155-5p blocks GC-MSC function in gastric cancer. Knockdown of miR-155-5p triggers BM-MSC to adopt GC-MSC-like functions.

### miR-155-5p underexpression promotes the transition of BM-MSC into GC-MSC-like cells via NF-κB p65 targeting

**Figure 4:**
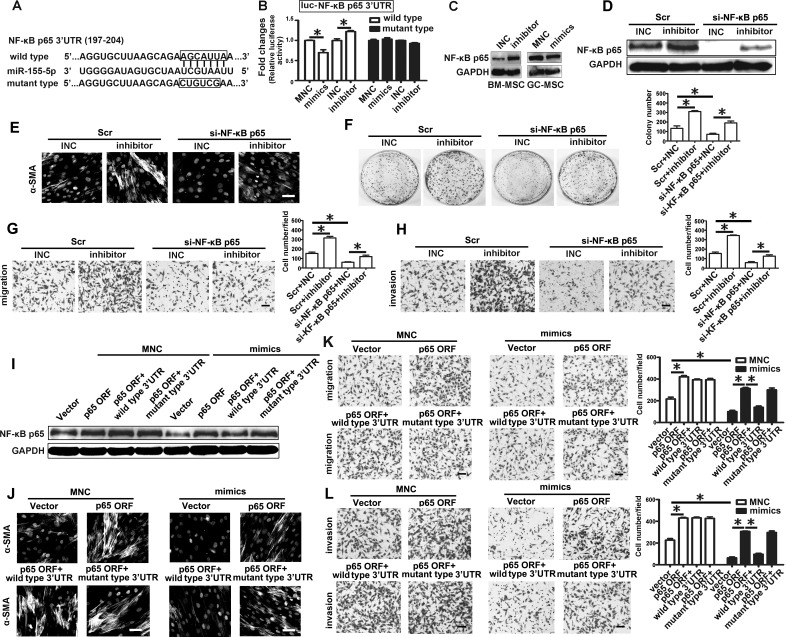
miR-155-5p inhibition triggers transition of BM-MSC to GC-MSC-like cells via NF-κB p65 targeting **A.** The position and sequences of the predicted binding sites complementary to the seed region of miR-155-5p in 3′UTR of NF-κB p65 mRNA are shown in black box. Wild type, luciferase reporter vector contains the sequences of the predicted binding sites. Mutant type, mutant the predicted binding sites with the sequence in the box. **B.** luciferase activity assay. **C.** Western blotting analysis of NF-κB p65 in miRNA inhibitor or mimics transfected MSCs. **D.**-**H.** Knock down of NF-κB p65 in BM-MSC before transfected with miR-155 inhibitor. Scr, Scramble control; si-NF-κB p65, siRNA against NF-κB p65. **I**.-**L**., NF-κB p65 ORF, ORF constructs with 3′-UTR (wild type) or 3′ UTR with mutated target site (mutant type) vectors were transfected separately with miR-155-5p mimics in BM-MSC. **D.** and **I.** NF-κB p65 protein detection. **E.** and **J.** Immunofluorescence assay of α-SMA. **F.** Colony formation assay. **G.** and **K.** Migration analysis. **H.** and **L.** Invasion assay. Data were presented as Means ±SD.*, *P* < 0.05.

NF-κB p65 was predicted as a potential target of miR-155-5p by miRTarBase and TargetScan software (Figure 4A). To elucidate the relationship between miR-155-5p and NF-κB p65, we generated 3′-untranslated regions (UTR) reporter vectors (wild-type, luc-NF-κB p65 3′-UTR) containing the predicted sequences. Luciferase activity assay showed that miR-155-5p mimics reduced the relative firefly luciferase activity, while miR-155-5p inhibitor remarkably increased the activity (Figure 4B). However, these changes did not occur when the predicted sites were mutated (mutant type, luc-NF-κB p65 3′-UTR) (Figure 4A and 4B). miR-155-5p inhibitor markedly increased NF-κB p65 protein level in BM-MSC. Conversely, miR-155-5p mimics decreased its expression level in GC-MSC (Figure 4C). These results indicate that NF-κB p65 is a target of miR-155-5p.

Further, we knocked down NF-κB p65 protein level by siRNA in BM-MSC before transfection with miR-155-5p inhibitor (Figure 4D). Immunofluorescence intensity of α-SMA was enhanced by miR-155-5p inhibitor in the control group, but this effect was abolished in the si-NF-κB p65 group (Figure 4E). The promotional effect of miR-155-5p inhibitor-transfected BM-MSC on the growth viability, migration and invasive capacities of HGC-27 cells were also blocked after knockdown of NF-κB p65 (Figure 4F-4H). Plasmids were constructed containing the NF-κB p65 open reading frame (ORF), ORF with wild type 3′-UTR or ORF with mutated target site in the 3′-UTR and transfected separately with miR-155-5p mimics in BM-MSC. NF-κB p65 protein levels overexpressed by the three NF-κB p65 ORF constructs were equivalent in the MNC groups. Compared to the MNC group, NF-κB p65 protein levels were reduced in the miR-155-5p mimics group. Among these constructs, the NF-κB p65 ORF with wild type 3′-UTR construct group exhibited the least level of NF-κB p65 protein in the mimic co-transfection group (Figure 4I). The phenotype and function of BM-MSC in the MNC group caused by the three NF-κB p65 ORF constructs were similar to those induced by miR-155-5p inhibitor. However, only the effect of NF-κB p65 ORF with wild type 3′-UTR construct on BM-MSC was suppressed after co-transfection with miR-155-5p mimics (Figure 4J-4L). These data suggest that NF-κB p65 is an important target of miR-155-5p. miR-155-5p inhibition promotes transition of BM-MSC to GC-MSC-like cells via NF-κB p65 targeting.

### miR-155-5p downregulation triggers the transition depending on NF-κB p65 activation

**Figure 5:**
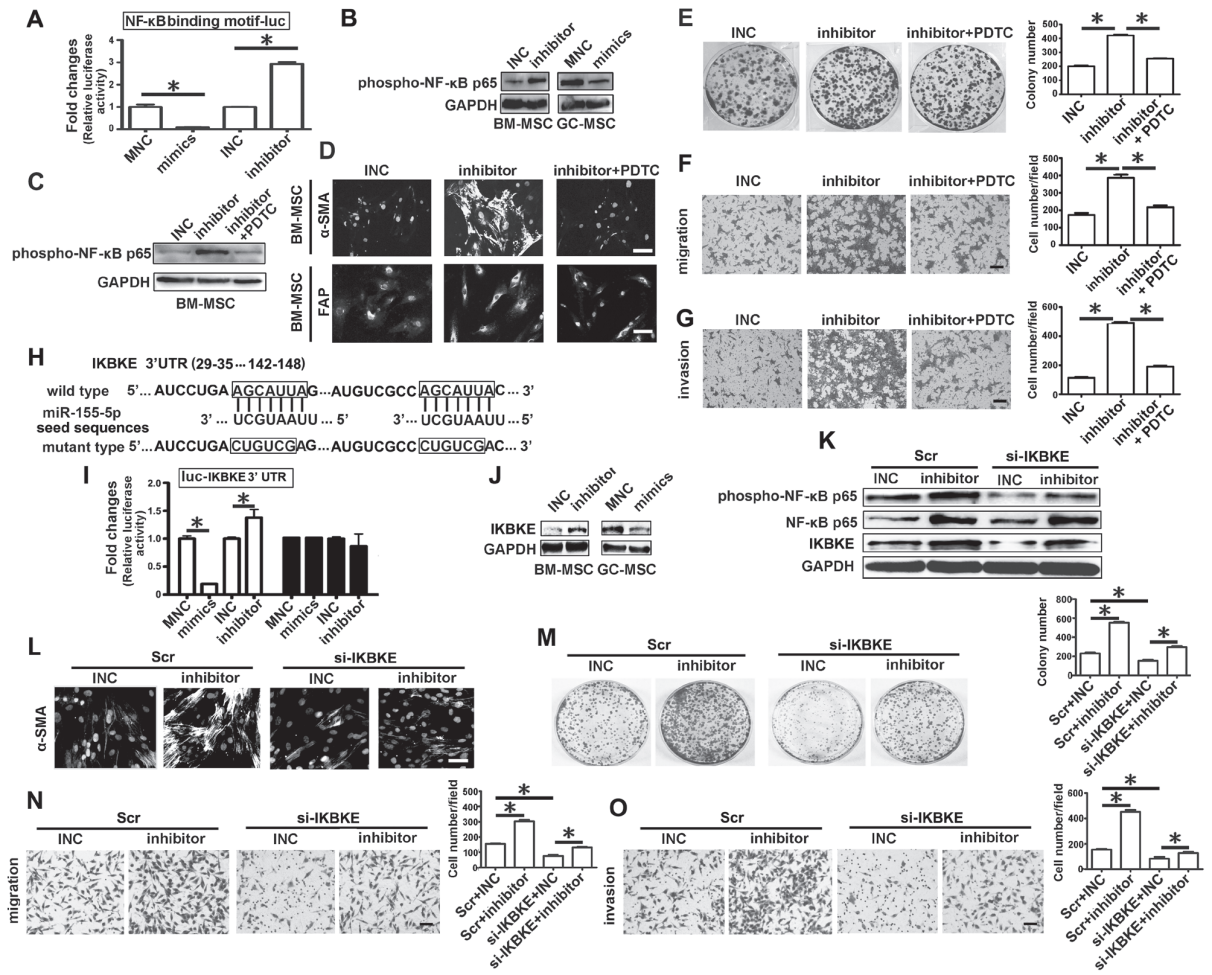
IKBKE validated as a target of miR-155-5p is involved in miRNA inhibitor activating NF-κB p65 **A.** Luciferase activity assay. NF-κB p65 binding motif were constructed into pGL3-Basic vector, which were used to test activation of NF-κB. **B.** Western blotting analysis of phospho-NF-κB p65 in miRNA inhibitor or mimics transfected MSCs. **C.**-**G.** BM-MSC were pretreated with NF-κB inhibitor PDTC for two hours, then transfected with miR-155 inhibitor for 48h. **H.**-**J.** IKBKE was validated as target gene of miR-155-5p. **K.**-**O.** Knock down of IKBKE in BM-MSC before transfected with miR-155 inhibitor. **C**. Western blotting analysis of phospho-NF-κB p65 after BM-MSC treated with PDTC. **D**. and **L**. α-SMA protein levels detection in BM-MSC; **E**. -**M**. Colony formation assay. **F**. and **N**. Migration analysis. **G**. and **O**. Invasion assay. **H**. The position and sequences of the predicted binding sites complementary to the seed region of miR-155-5p in 3′UTR of IKBKE mRNA are shown in black box. **I**. luciferase activity assay. **J**. Western blotting analysis of IKBKE in miRNA inhibitor or mimics transfected MSCs. K, Detection of IKBKE, phospho-NF-κB p65 and NF-κB p65 protein. Data were presented as Means ±SD.*, *P* < 0.05.

We constructed an NF-κB elemental response luciferase reporter (NF-κB binding motif-luc) to evaluate the effect of miR-155-5p on the activation of NF-κB. Luciferase reporter assays revealed that the miR-155-5p inhibitor activated NF-κB, while miR-155-5p mimics suppressed NF-κB activation (Figure 5A). NF-κB p65 was significantly phosphorylated in the presence of the miRNA inhibitor in BM-MSC, whereas it was de-phosphorylated by miRNA mimics in GC-MSC (Figure 5B). As shown in Supplementary Figure 2, phospho-NF-κB-p65 levels were altered in the same way as NF-κB-p65 protein level in BM-MSC co-transfected with the three kinds of NF-κB p65 ORF constructs and miR-155-5p mimics. These data suggest that miR-155-5p downregulation not only increased NF-κB p65 protein level, but also stimulated its activation.

Then we used the NF-κB inhibitor PDTC (pyrrolidine dithiocarbamic acid) to treat BM-MSC before transfection with the miRNA inhibitor. PDTC treatment eliminated the effect of the miR-155-5p inhibitor on NF-κB p65 activation (Figure 5C). The immunofluorescence intensity of α-SMA and FAP induced by the miR-155-5p inhibitor in BM-MSC was obviously suppressed by PDTC (Figure 5D). The number of HGC-27 cell colonies in the miRNA inhibitor-transfected BM-MSC group was reduced by PDTC to a similar extent to that in the INC control group (Figure 5E). The miR-155-5p inhibitor-enhanced migrating and invasive capacities of HGC-27 cells were also abolished by PDTC (Figure 5F and 5G).

IKBKE (IKKi/IKKε), a non-canonica IκB kinase, has two predicted target sites in 3′-UTR matched to the seed sequences of miR-155-5p (Figure 5H). Luciferase activity assay showed that miR-155-5p mimics reduced relative firefly luciferase activity, while miR-155-5p inhibitor remarkably increased this activity (Figure 5I). However, these changes did not occur when the predicted sites were mutated (Figure 5I). IKBKE protein level was increased in BM-MSC after transfection with miR-155-5p inhibitor but was reduced in GC-MSC treated with miR-155-5p mimics (Figure 5J). NF-κB p65 phosphorylation level in the miR-155-5p inhibitor group was reduced by si-IKBKE to the level observed in the INC and scramble (Scr) co-transfection control groups, but NF-κB p65 protein levels were not affected (Figure 5K). IKBKE suppression could also abolish the effect of miR-155-5p inhibitor on the transition of BM-MSC to GC-MSC-like cells (Figure 5L-5O). The data suggest that miR-155-5p inhibitor may activate NF-κB p65 by targeting upregulation of IKBKE. Thus, the function of miR-155-5p inhibitor in BM-MSC is dependent on NF-κB p65 activation.

### IKBKE, NF-κB p65 and phospho-NF-κB p65 proteins expression in MSC-like cells of gastric cancer tissues

**Figure 6:**
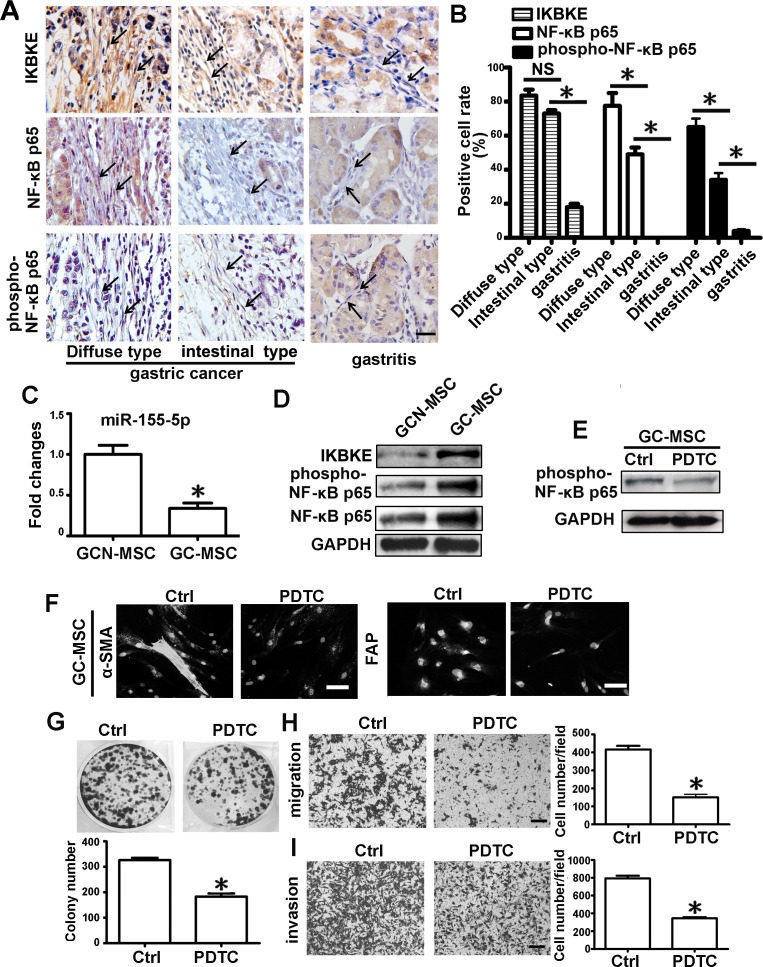
NF-κB p65 protein is enriched and activated in MSC-like cells of gastric cancer tissues **A.** IKBKE, NF-κB p65 and phospho-NF-κB p65 analysis by immunochemistry in diffuse type of gastric cancer, intestinal type of gastric cancer and gastritis tissues. Representative images were shown. Stroma cells with elongated and spindle shape are recognized as MSC-like cells. Representative cells are indicated by arrows. **B.** The percentage of the positive cells among these specified cells in two types of gastric cancer tissues and gastritis tissues were presented as columns. **C.**-**I.** GC-MSC and GCN-MSC were isolated from gastric cancer tissues and adjacent non-cancerous gastric tissues. **C.** *q*RT-PCR of miR-155-5p expression levels in GC-MSC versus GCN-MSC. **D.** Western blotting analysis of IKBKE, phospho-NF-κB p65 and NF-κB p65 protein in GC-MSC and GCN-MSC. **E.** Western blotting analysis of phospho-NF-κB p65 in GC-MSC after treated with PDTC. **F.** Immunofluorescence assay of α-SMA and FAP protein levels in GC-MSC. **G.** Colony formation assay. **H.** Migration analysis. **I.** Invasion assay. Data were presented as Means ±SD.*, *P* < 0.05.

Two types of metastatic gastric cancer tissues and gastritis tissues were randomly selected for IKBKE, NF-κB p65 and phospho-NF-κB p65 proteins analysis by immunohistochemistry. For lack of specific markers for MSCs, the elongated and spindle-shaped cells surrounding the gastric epithelium were defined as MSC-like cells. Compared to the intestinal type of gastric cancer tissues, these proteins positive intensity were obviously stronger in the MSC-like cells of the diffuse type (Figure 6A). The percentage of positively stained cells for IKBKE, NF-κB p65 and phospho-NF-κB p65 in the diffuse type of gastric cancer were up to 83.5%, 80.8% and 65.0%, respectively, but the positive cell percentages of the latter two in the intestinal type of gastric cancer were reduced to 49.5% and 34.0 %. However, there was no difference in the positive cell percentages of IKBKE between two types of gastric cancer (Figure 6B). As to gastritis tissues, MSC-like cells mostly stained negative. The rate of IKBKE, NF-κB p65 and phospho-NF-κB p65 positivity in gastritis tissues were just 19.3%, 0% and 4%, respectively (Figure 6B).

### NF-κB p65 activation sustaining the tumor-promoting phenotype and function of GC-MSC

We isolated GC-MSC and GCN-MSC from gastric cancer tissues and adjacent non-cancerous tissues (Supplementary Figure 1). Compared to GCN-MSC, miR-155-5p level was significantly downregulated with upregulation of IKBKE, NF-κB p65 and phospho-NF-κB p65 protein levels in GC-MSC (Figure 6C, 6D). Inactivation of NF-κB p65 by PDTC significantly inhibited α-SMA and FAP expression in GC-MSC (Figure 6E, 6F), and greatly suppressed the capacity of GC-MSC to promote gastric cancer cell HGC-27 colony formation, migration and invasion (Figure 6G-6I).

NF-κB p65 is a well-known transcription factor and its activation is related to cytokine expression. *q*RT-PCR detection showed that IL-6, IL-8 and MCP-1 expression levels were significantly reduced in GC-MSC after PDTC treatment (Supplementary Figure 3A). Knockdown of each cytokine by siRNA in GC-MSC slightly inhibited α-SMA and FAP expression and the effect of GC-MSC on HGC-27 cell migration capacity (Supplementary Figure 3B-3D). However, we used the siRNA pool to knock down all three cytokines, which significantly suppressed immunofluorescence intensity of α-SMA and FAP and abolished the role of GC-MSC in promoting HGC-27 migration (Supplementary Figure 3C, 3D). These results indicate that as downstream molecules of NF-κB p65, IL-6, IL-8 and MCP-1 work cooperatively to sustain the tumor-promoting phenotype and function of GC-MSC.

## DISCUSSION

Recently, tumor associated MSC were sequentially reported to be isolated from many kinds of solid tumors [21, 22, 23]. A series of relevant studies explored the origins of tumor stroma cells and found that BM-MSC might be the precursors of most of the stroma cells in the tumor microenvironment [24]. In the present study, we found that GC-MSC, as an important type of gastric cancer associated stroma cells, show different phenotype and function from those of BM-MSC. Knockdown of miR-155-5p induced BM-MSC to acquire a GC-MSC-like phenotype and function through NF-κB activation.

Previous studies have uncovered that miR-155-5p plays an important role in recruiting normal cells to the tumor microenvironment. Wang *et al.* and Chen *et al.* demonstrated that miR-155-5p deficiency promotes solid tumor growth by increasing the recruitment of myeloid-derived suppressor cells (MDSCs) to the tumor microenvironment [25, 26]. Moreover, miR-155-5p was involved in reprogramming normal fibroblasts into CAFs in ovarian cancer [19]. Yu *et al.* revealed that miR-155-5p-deficient bone marrow accumulates more macrophages in the tumor microenvironment and polarizes the macrophages into the M2 tumor promoting type [27]. Thus, we focused on a possible link between miR-155-5p and the transition of BM-MSC into GC-MSC in gastric cancer. Compared to BM-MSC, miR-155-5p was significantly downregulated in GC-MSC. Knockdown of miR-155-5p in BM-MSC induced the acquisition of a GC-MSC-like phenotype and function, while overexpression of miR-155-5p reversed the tumor-promoting phenotype and function of GC-MSC. These data suggest that miR-155-5p is also involved in reprogramming BM-MSC into GC-MSC in gastric cancer and might be therapeutic target for gastric cancer.

A large number of studies have demonstrated that miR-155-5p plays a multifunctional role in human cancer cells by regulating many target genes. Several research groups have reported that miR-155-5p acts as an oncomiR by downregulating tumor suppressor genes, including TP53INP1 [28], CDC73 [29] and DMTF1 [30]. However, other studies suggested that miR-155 may act as a tumor suppressor in certain solid tumors by targeting tumor oncogenes such as claudin-1[31] and c-Myc [32]. miR-155-5p was upregulated in CAFs, but downregulated in GC-MSC, which suggests that miR-155-5p may act as promoter or suppressor in remodeling tumor stroma via regulation of different target genes. We predicted and validated NF-κB p65 as a target of miR-155-5p in our system, which is consistent with previous finding [33]. It has been widely acknowledged that NF-κB signaling pathway contributes to cancer through promotion of the tumor microenvironment [34]. Grégoire *et al.* reported that tumor-associated neutrophils activated other stroma cells in an NF-κB dependent manner in germinal center B-cell lymphomas [35]. Gain- and loss-of-function studies revealed that miR-155-5p was involved in the transition of BM-MSC to GC-MSC-like cells via targeting NF-κB p65. A previous study claimed that miR-155-5p mediated pancreatic cancer derived microvesicles reprogramming normal adjacent fibroblasts into CAF by downregulation of TP53INP1 protein levels [20]. Take together, these information implies that deregulation of miR-155-5p in GC-MSC has distinctive remodeling mechanisms from the other stromal cells in the tumor microenvironment.

As is well-known, phosphorylation is activating mechanism regulating NF-κB's function as a transcription factor. We found that miR-155-5p inhibitor not only induced NF-κB p65 expression, but also stimulated its activation. NF-κB inactivation by PDTC suppressed the effect of miR-155-5p inhibition on the transition of BM-MSC to GC-MSC. These data indicate that miR-155-5p inhibitor might function in this transition finally activating NF-κB p65. As shown, the level of phospho-NF-κB-p65 was increased in BM-MSC after transfected with the three NF-κB ORF constructs, which provides an explanation why overexpresseion of NF-κB p65 promoted the conversion of BM-MSC to GC-MSC-like cells. The effect of NF-κB p65 ORF with wild type 3′-UTR construct on BM-MSC inhibited by miR-155-5p mimics may be attributed to NF-κB p65 inactivation. The role of miR-155-5p inhibitor that was abolished by si-NF-κB-p65 in BM-MSC may be also resulted from reduction in NF-κB-p65 activation. Moreover, we predicted and validated IKBKE as another target gene of miR-155-5p. Several studies demonstrated that IKBKE is an important mediator of the activation of NF-κB and supported a role for IKBKE-mediated Ser536 phosphorylation of the p65 NF-κB subunit [36, 37]. knockdown of IKBKE reduced the level of NF-κB p65 phosphorylation, but didn't affect the protein level of NF-κB p65 induced by the miR-155-5p inhibitor. This suggests that miR-155-5p inhibitor activates NF-κB p65 via IKBKE. IKBKE inhibition still exerted the same effect as NF-κB knockdown on miR-155-5p inhibitor in BM-MSC, which further supports the conclusion that NF-κB p65 activation is the key signaling mechanism mediating miR-155-5p inhibition promoting transition of BM-MSC into GC-MSC-like cells.

Clinical gastric tissues detection revealed that NF-κB p65 signaling was highly enriched and activated in stroma cells of gastric cancer and related to the pathological progression of gastric cancer. Consistent with the prior findings, miR-155-5p was downregulated and inversely correlated with IKBKE level, NF-κB p65 levels and activation in GC-MSC. Inactivation of NF-κB p65 by PDTC significantly reversed the phenotype and function of GC-MSC. As inflammatory tumor microenvironment including cytokines are orchestrated by NF-κB [38], we identified IL-6, IL-8 and MCP-1 as important downstream moleculars of NF-κB p65 for sustaining the phenotype and function of GC-MSC. These data indicated that NF-κB p65 activity in tumor stroma cells can not only be used to reflect pathological progression of gastric cancer, but also be served as therapeutic target for gastric cancer.

miR-155-5p perturbation led BM-MSC to acquire a tumor-promoting function, but their phenotype and function did not fully reach those of GC-MSC. Additional deregulated miRNAs in GC-MSC need to be identified and their potential role in reprogramming BM-MSC to GC-MSC needs to be elucidated. Previous studies claimed that miR-155-5p could recruit normal cells to the tumor microenvironment. Whether miR-155-5p is participated in recruiting BM-MSC to gastric cancer and promoting gastric cancer microenvironment formation need to be explored in the future.

## MATERIALS AND METHODS

### Cell lines and clinical tissues

Human BM-MSC, GC-MSC and GCN-MSC were isolated, cultured and characterized as described previously [7, 8]. Gastric cancer cell line HGC-27 and embryonic kidney 293T were purchased from Cell Bank, Chinese Academy of Sciences (Shanghai, China). All cells were cultured in Dulbecco's Modified Eagle Medium (DMEM, Gibco, USA) supplemented with 10% fetal bovine serum (FBS, Gibco) at 37°C in humidified air with 5% CO_2_. MSC conditioned medium preparation: 5×10^5^ of MSC were planted in 10 cm dish. Cell culture medium was harvested after 48h, centrifuged at 1500 rpm for 10 min, then filtered through a 0.22μm membrane (Millipore, Germany) and stored in −80°C until use. PDTC treatment: PDTC as a NF-κB inhibitor were dissolved in PBS (Phosphate Buffered Saline). BM-MSC were pretreated with 100 nM PDTC for two hours before transfection. GC-MSC were directly treated with the same concentration of PDTC (Sigma-Aldrich Co. LLC., USA). Paraffin sections of gastric cancer and gastritis tissues were collected from the Affiliated Peoples' Hospital of Jiangsu University. Documented informed consent was obtained from all subjects and the Ethics Committee of Jiangsu University approved all aspects of the study.

### RNA isolation and *q*RT-PCR

Total RNA of MSCs were extracted with Trizol Reagent (Invitrogen, USA). miRNA *q*RT-PCR were performed with miScript II RT Kit and miScript SYBR Green PCR Kit (Qiagen, Germany). *q*RT-PCR of cytokine mRNAs were conducted with HiScript 1st Strand cDNA Synthesis Kit and SYBR-Green I Real-Time PCR kit (Vazyme Biotech Co., Ltd, China). The amplification fluorescence signals were detected by CFX96 Touch™ Real-Time PCR Detection System (Bio-Rad, USA). The relative expression levels of miRNAs and mRNAs were normalized to the expression of RNU6B and β-actin, respectively. miRNA primers were supplied by Qiagen. All the primers sequences and *q*RT-PCR conditions for mRNAs are listed in Supplementary Table 1.

### Immunofluorescence

The primary antibody against α-SMA (BS70000, Bioworld Technology, Inc., USA) and FAP (ab53066, Abcam, USA) were used. MSCs were incubated with the primary antibodies at 4°C overnight and followed by Cy3-conjugated anti-rabbit secondary antibodies (Invitrogen, USA). Finally, the cells were then stained with Hoechst33342 for nuclear staining, and the images were acquired with a microscope (Olympus, Japan).

### Colony forming assay

HGC-27(1×10^3^) were seeded and attached into a 6-well plate overnight. The MSCs conditioned medium was added and changed in a two-day interval for eight days. The colony were fixed with 4% paraformaldehyde, stained with crystal violet, then photographed and counted.

### Transwell migration and invasion assay

MSC conditioned medium was added in the bottom chambers of the transwell plates. 1.5×10^5^ of HGC-27 were planted into the top chambers (8-μm pore size, Corning, USA), and incubated for 10 h for migration assay. For invasion assay, 2×10^5^ of HGC-27 were planted into the top chambers pre-coated with matrigel (BD Biosciences, USA), and incubated for 12 h. The migration or invasion cells was photographed and counted under a microscope at least 6 fields for each assay.

### Oligonucleotides transfection

miR-155-5p inhibitor, inhibitor negative control (INC), miR-155-5p mimics, mimics negative control (MNC), scramble control (Scr) and siRNA (against NF-κB p65, IKBKE, IL-6, IL-8 and MCP-1) were synthesized and purified by Genepharma. Transfection was performed using Lipofectamine 2000 (Invitrogen, USA), and the concentration of inhibitor, mimics and siRNAs were 50 nM, 5 nM and 100 nM, respectively. siRNA pool containing a ratio of 1:1:1 of si-IL6, si-IL8 and si-MCP-1 was transfected at the concentration of 100 nM. After transfection for 48h, MSCs conditioned medium were harvested and prepared. Total RNA and protein were isolated from MSCs as described previously [10]. Sequences and modifications of the oligonucleotides are shown in Supplementary Table 2.

### Western blotting

The primary antibodies against phospho-NF-κB p65 (No.3033), NF-κB p65 (No.3034) and IKBKE (No.2690) were purchased from Cell Signaling Technology. After incubation with the secondary antibodies (Bioworld Technology, Inc., USA), the signal was visualized using HRP substrate (Millipore, Germany) and analyzed using MD Image Quant Software. GAPDH was used as the loading control.

### Animal model

The animal studies were performed with approval of the University Committee on Use and Care of Animals of Jiangsu University. HGC-27 (1×10^6^) suspended in MSCs conditioned medium were subcutaneously co-injected into the flank of 5-week-old BALB/c nude mice (*n* = 6 for each group). Tumors were surgically removed 20 days after injection, photographed and weighted. Tumor volume was assessed by caliper measurement and calculated by the formula (L×W×W/2), where L represents length, and W represents width.

### Vector construction and Luciferase activity assay

The 3′ untranslated regions (3′-UTR) of NF-κB p65 and IKBKE mRNA were obtained by PCR and ligated into the pmirGLO dual-luciferase miRNA target expression vector (Promega, Madison, WI, USA). The mutant constructs were generated using Quik Change II Site-Directed Mutagenesis Kit (Agilent Technologies, Santa Clara, CA, USA). NF-κB p65 ORF (HG12054-UT) and control vector (G08AU6M9) were purchased from Sino Biological lnc.. NF-κB p65 ORF construct with wild type 3′-UTR or 3′-UTR with mutated target site were constructed as the corresponding luciferase reporter vectors. All the primer sequences are listed in Supplementary Table 3. NF-κB binding motif (5′-GGGAATTTCCGGGAATT TCCGGGAATTTCCGGGAATTTCC-3′) were synthesized and inserted into pGL3-Basic vector (NF-κB binding motif-luc). The activity of firefly luciferase was measured using a Dual-Luciferase Reporter Assay System (Promega, USA) and normalized to that of Renilla luciferase.

### Immunohistochemistry

The protein levels of IKBKE, NF-κB p65 and phospho-NF-κB p65 in formalin-fixed paraffin-embedded gastric cancer tissues and gastritis tissue were detected by immunohistochemistry. Briefly, the sections were incubated with primary antibody and secondary antibody, visualized with 3,39-diaminobenzidine (DAB) and then counterstained with hematoxylin for examination by the microscope. Fifteen diffuse type of gastric cancer tissues, eighteen intestinal type of gastric cancer tissues and thirteen gastritis tissues were chosen randomly and the positive cell rates were photographed and counted under a microscope at least six fields for each tissue.

### Statistical analysis

All experiments were conducted at least in triplicate. Data were presented as Means ±SD. Statistical analysis of the data was performed using Graph-Pad Prism 5 software. Differences between groups were analyzed by student's *t* test or one way ANOVA. *P* value < 0.05 is considered to be significant.

## SUPPLEMENTARY MATERIAL FIGURES AND TABLES


