# Supplementary material for: miR-155-5p inhibition promotes the transition of bone marrow mesenchymal stem cells to gastric cancer tissue derived MSC-like cells via NF-κB p65 activation
Source: Oncotarget. 2016 Feb 26;7(13):16567–80. doi: 10.18632/oncotarget.7767 (PMC4941335; doi:10.18632/oncotarget.7767)
Supplement: Supplementary file 1 [file oncotarget-07-16567-s001.pdf]

miR-155-5p inhibition promotes the transition of bone marrow mesenchymal stem cells to gastric cancer tissue derived MSC-like cells via NF-κB p65 activation

Supplementary Material

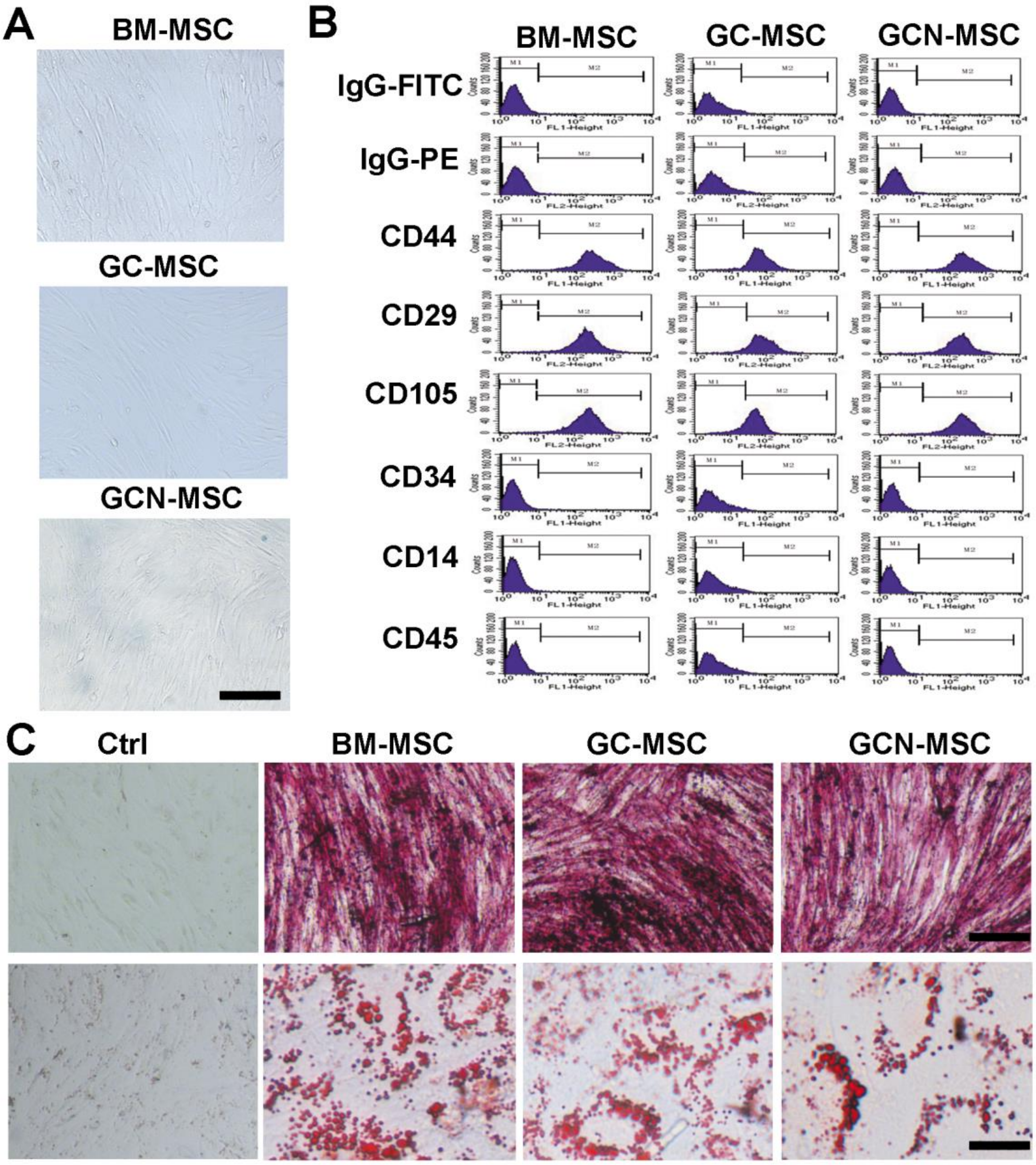

Supplementary Figure 1: Isolation and characteristics of mesenchymal stem cells

A. Morphological appearance of bone marrow mesenchymal stem cells (BM-MSC), gastric cancer tissues derived MSC-like cells (GC-MSC) and adjacent non-cancerous gastric tissues derived MSC-like cells (GCN-MSC) in the 3rd passage. Magnification: ×100, *Scale bar*=50μm. B. Flow cytometric analysis of the surface

antigens of BM-MSC, GC-MSC and GCN-MSC. Monoclonal antibodies against CD44, CD34, CD14 and CD45 were FITC-conjugated, while CD29 and CD105 were PE-conjugated. IgG-PE and IgG-FITC were used in the control group. C. Differentiation potential of BM-MSC, GC-MSC and GCN-MSC. MSCs grew in adipogenic or osteogenic medium for two weeks. Upper: Alkaline phosphatase staining detection for osteogenic differetiantion; Lower: Oil-Red-O staining detection for adipogenic differentiation. Magnification: ×100, *Scale bar* = 50μm.

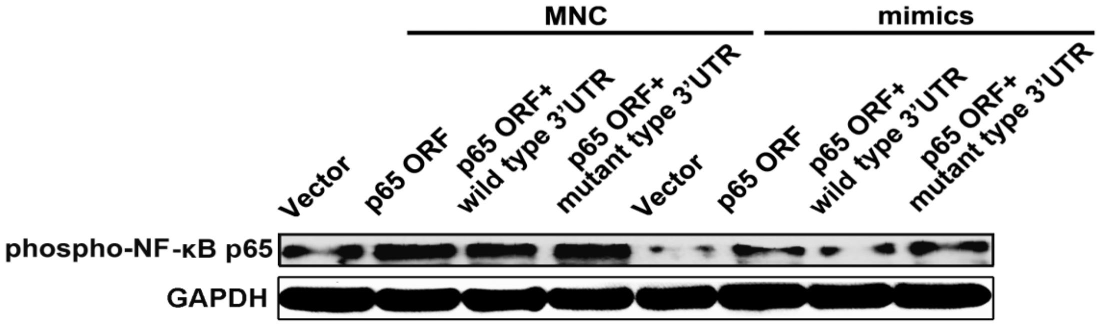

Supplementary Figure 2: Phosphorylation level of NF-κB p65 detection in BM-MSC co-transfected with ORF constructs and miR-155-5p mimics

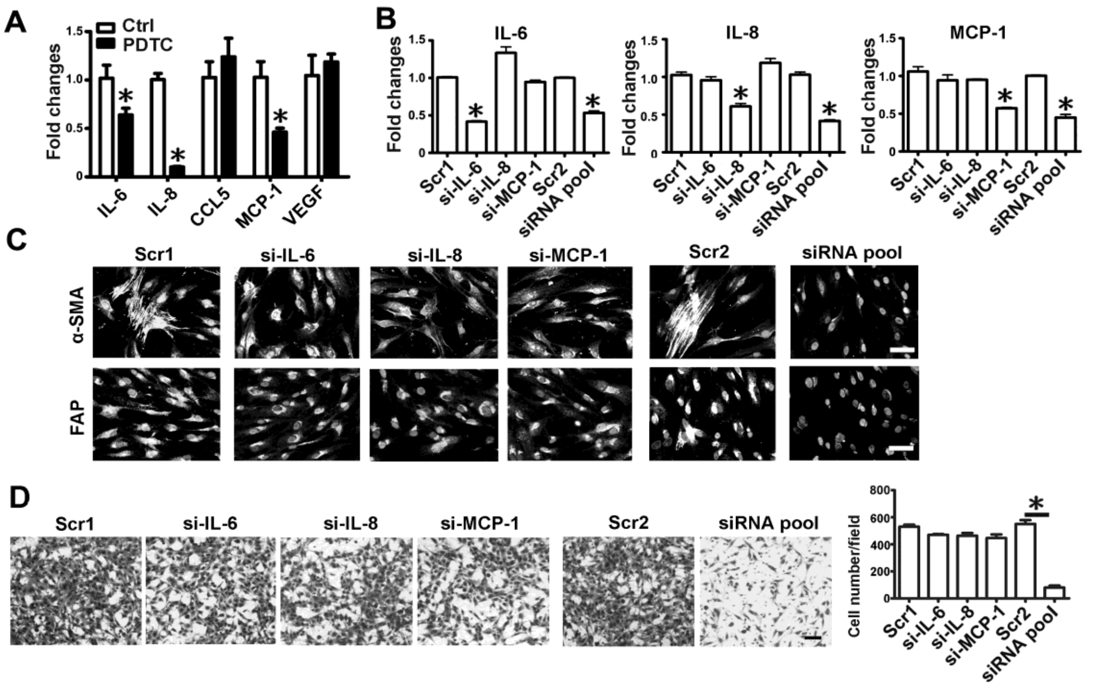

Supplementary Figure 3: Downstream molecules of NF-κB p65 identification and their function analysis in GC-MSC

A. *q*RT-PCR of cytokine mRNA expression in GC-MSC after treated with PDTC. B.-D. GC-MSC was transfected with siRNA against each cytokine separately or siRNA pool. B, *q*RT-PCR of IL-6, IL-8 and MCP-1 mRNA expression. C. α-SMA and FAP protein levels detected by immunofluorescent staining, Magnification: 200×, *Scale bar* = 50μm. D. Migration analysis. Magnification: 100×, *Scale bar* =50μm. Representative graphs were shown. Cell number of each field were counted and presented as columns. Data were presented as Means ±SD. * , $P < 0.05$.

Supplementary Table 1: Primers sequences for *q*RT-PCR and the conditions of amplification

Genes	Primers sequences (5'-3')	Annealing Temperatures(℃)	Product length(bp)
β-actin	For[a]:CACGAAACTACTCCCAACTCC	56	265
	Rev[b]:CATACTCC TGCTTGAGCTGATC		
IL-6	For[a]: TACATCCTCGACGGCATCTC	61	252
	Rev[b]: AGCTCTGGCTTGTTCCTCAC		
IL-8	For[a]:GCTCTGTGTGAAGGTGCAGTTT	62	144
	Rev[b]:TTCTGTGTTGGCGCAGTGT		
CCL5	For[a]:GGATTCCTGCAGAGGATCAA	62	154
	Rev[b]:GTGGTGTCCGAGGAATATGG		
MCP-1	For[a]:GAACCGAGAGGCTGAGACTA	59	259
	Rev[b]:GCCTCTGCACTGAGATCTTC		
VEGF	For[a]: CCTTGCTGCTCTACCTCCAC	58	280
	Rev[b]:ATCTGCATGGTGATGTTGGA		

[a] For, forward primer; [b] Rev, reverse primer. h, human

Supplementary Table 2: Sequences and modifications of the oligonucleotides

oligonucleotide	Sequences(5'-3')	modification
Mimics negative control (MNC)	Sense:UUCUCCGAACGUGUCACGUTT<br>Antisense:ACGUGACACGUUCGGAGAATT	2'-O-Methyl-modified
miR-155-5p mimics	Sense:UUAAUGCUAAUCGUGAUAGGGGU<br>Antisense:CCCUAUCACGAUUAGCAUUAAUU	
Inhibitor negative control (INC)	CAGUACUUUUGUGUAGUACAA	2'-O-Methyl-modified
miR-155-5p inhibitor	ACCCCUAUCACGAUUAGCAUUAA	
Scramble control （Scr）	Sense:UUCUCCGAACGUGUCACGUTT<br>Antisense:ACGUGACACGUUCGGAGAATT	2'-O-Methyl-modified
si-NF-κB p65	Sense:CCUCCUUUCAGGAGAUGAATT<br>Antisense:UUCAUCUCCUGAAAGGAGGTT	
si-IKBKE	Sense:GCUGAACCACCAGAACAUUTT<br>Antisense:AAUGUUCUGGUGGUUCAGCTT	
si-IL6	Sense:CCCAGGAGAAGAUUCCAAATT<br>Antisense:UUUGGAAUCUUCUCCUGGGTT	
si-IL8	Sense:GCCAGAUGCAAUACAAGAUTT<br>Antisense:AUCUUGUAUUGCAUCUGGCTT	
si-MCP-1	Sense:GCUGUUAUAACUUCACCAATT<br>Antisense:UUGGUGAAGUUAUAACAGCTT	

Supplementary Table 3: Primers sequences for vector construction

Vector	Primers sequences (5'-3')
luc-NF-κB p65 3'UTR wild type	For[a]: GAAGCCCTCCAAAAGCACTTAC<br>Rev [b]: CTAGCCAGCTTGGCAACAGAT
luc-NF-κB p65 3'UTR mutant type	For[a]:tctctttttggaggtgcttaagcaga***ctgtcg***aacttctctggaaaggggggagctgg<br>Rev[b]:ccagctcccccctttccagagaagtt***cgacag***tctgcttaagcacctccaaaaagaga
luc-IKBKE p65 3'UTR wild type	For[a]: TGAAGCTGCTGGCATCTGAC<br>Rev [b]: ATGGAGGAGCAGTACCTGAG
luc-IKBKE p65 3'UTR mutant type	For[1a]:gctccatggggcacatgaggcatcctga***ctgtcg***agaatgattccaacactgctcttc<br>Rev[1b]:gaagagcagtgttggaatcattct***cgacag***tcaggatgcctcatgtgccccatggagc<br>For [2a]: gctgctggccaggatgtcgccc***ctgtcg***accttccactgcctttctccc<br>Rev[2b]:gggagaaaggcagtggaaggt***cgacag***ggcgacatcctggccagcagc

[a] For, forward primer;[b] Rev, reverse primer; [c] Sequences in italic and bold indicate mutant sites.
